# Prevalence of Human Toxoplasmosis in Spain Throughout the Three Last Decades (1993–2023): A Systematic Review and Meta-analysis

**DOI:** 10.1007/s44197-024-00258-w

**Published:** 2024-06-12

**Authors:** Mariola Miguel-Vicedo, Paula Cabello, M. Carmen Ortega-Navas, David González-Barrio, Isabel Fuentes

**Affiliations:** 1 Toxoplasmosis and Protozoosis Unit, Parasitology Reference and Research Laboratory, Spanish National Centre for Microbiology, Health Institute Carlos III, Majadahonda, Madrid, Spain; 2 Department of Educational Theory and Social Pedagogy, National University of Distance Education (UNED) Madrid, Madrid, Spain; 3 International University of Valencia-VIU, 46002 Valencia, Spain; 4 Ph.D. Program in Biomedical Science and Public Health. IMIENS, National University of Distance Education (UNED) Madrid, Madrid, Spain

**Keywords:** *Toxoplasma gondii*, Toxoplasmosis, Prevalence, Seroprevalence, Spain, Human, HIV patient, Cerebral toxoplasmosis, Pregnant woman

## Abstract

Humans are infected by *Toxoplasma gondii* worldwide and its consequences may seriously affect an immune deprived population such as HIV and transplanted patients or pregnant women and foetuses. A deep knowledge of toxoplasmosis seroprevalence in Spain is needed in order to better shape health policies and educational programs. We present the results of the first systematic review and meta-analysis on the human prevalence for this disease in Spain. Databases (PubMed, Web of Science, SCOPUS and Teseo) were searched for relevant studies that were published between January 1993 and December 2023 and all population-based cross-sectional and longitudinal studies reporting the human seroprevalence in Spain were revised. Within the population analysed, our targeted groups were immunocompetent population, pregnant women and immunocompromised patients. Among 572 studies and 35 doctoral theses retrieved, 15 studies and three doctoral theses were included in the meta-analysis. A random effects model was used for the meta-analyses due to the high heterogeneity found between studies (*I*^2^: 99.97), since it is a statistically conservative model, in addition to allowing better external validity. The global pooled seroprevalence was 32.3% (95% CI 28.7–36.2%). Most of the studies carried out were in pregnant women and the meta-analysis reported that the pooled seroprevalence of toxoplasmosis in pregnant women in Spain was 24.4% (24,737/85,703, 95% CI 21.2–28.0%), based on the random effects model. It is recommended to continue monitoring the seroprevalence status of *T. gondii* in order to obtain essential guidelines for the prevention and control of the infection in the population.

**Supplementary Information:**

The online version contains supplementary material available at 10.1007/s44197-024-00258-w.

## Introduction

*Toxoplasma gondii* is a protozoan isolated for the first time in 1908 [1]. It is postulated that the parasite had its origin in South America and it was spread through transatlantic migrations of cats, mice and rats as a consequence of slavery trading [2]. All warm-blooded animals and humans are susceptible to infection by this parasite. *Toxoplasma gondii* has been studied deeply due to its importance medically and veterinary being an important zoonosis in the One Health approach [3]. The average global human seroprevalence rate for this disease is estimated to be 25.7% although this rate presents variations depending on a wide variety of factors such as geographical, economical or environmental [4].

Regarding the life cycle of the *Toxoplasma gondii*, there are three known stages that can infect cells: (*i*) tachyzoite (found in acute infections) (*ii*) bradyzoite (characteristic of the chronic infections) and (*iii*) sporozoite (developed inside the oocysts, produced only in the definitive host). *Toxoplasma gondii* infection occurs in birds and terrestrial and aquatic mammals. These animals are considered intermediate hosts of *T. gondii* since the asexual stage of the parasite develops in them. Members of the family Felidae present the sexual stages, considering them the definite hosts [5, 6]. There are many factors that can lead to contracting the disease including undercooking meat, consuming raw or fresh vegetables not properly sanitised or blood transfusion [5]. The infection usually occurs in the majority of immunocompetent individuals asymptomatically or with mild symptoms such as flu, Q fever or haematological alterations, [7, 8] although in immunocompetent individuals with acute toxoplasmosis, severe cases may occur with pulmonary symptoms or involvement of various organs, in many of them related to the virulence of the parasite genotype involved. Different studies indicate that latent Toxoplasma infection, in the chronic phase, is responsible for neurological disorders [9–11]. After the acute infection, the parasite persists in cysts in different muscles and organs, controlled by the specific immunity originated as a response, therefore specific IgG anti-*Toxoplasma* antibodies will be detected for life. Changes in the immune status can cause the reactivation of the parasites located in the cysts, causing serious pathologies. Must be highlighted that some specific groups of patients are at risk for severe infections, including pregnant women, children with congenital toxoplasmosis and immunocompromised patients [11–14]. In immunocompromised patients such as AIDS or organ-transplanted population, toxoplasmosis is a frequent and serious opportunistic infection that usually happens as a result of reactivation of chronic infection. In these patients, clinical symptoms consist of mental status changes, seizures, sensory abnormalities, cerebellar signs, movement disorders, and neuropsychiatric findings and extensive and generalised manifestations that can cause death [15, 16]. Contracting toxoplasmosis during pregnancy can potentially have dangerous consequences or even be lethal in the foetus due to the possibility of intrauterine infection and consequently, develop congenital toxoplasmosis [17]. Routine prenatal serological screening throughout pregnancy is important for early diagnosis and treatment during pregnancy; however, despite its importance, some countries do not implement prenatal screening programmes [17].

Studying the toxoplasmosis seroprevalence rate in Spain and its evolution to have a precise estimation of this value can help health-care professionals to control this disease and avoid its consequences and assist health authorities to take action on this serious public health issue, such as implementing prenatal screening and treatment programs. For example, knowing the prevalence in pregnant women can contribute to a better understanding of the risk and the overall exposure rate. With this purpose, a systematic review was performed in order to evaluate the seroprevalence rate of toxoplasmosis in Spain throughout the last three decades (1993–2023).

## Materials and Methods

### Search Strategies and Selection Criteria

The systematic review and meta-analysis in full accordance with the Preferred Reporting Items for Systematic Reviews and Meta‑Analyses (PRISMA) guidelines [18] were used to conduct the current study. The databases used in this research were PubMed (MEDLINE, National Library of Medicine, USA), Web of Science (Thomson Reuters, USA) and Scopus (Elsevier, The Netherlands) for all the studies that contained information regarding toxoplasmosis seroprevalence in humans from January 1993 to December 2023 from Spain. In addition, the Ordered Spanish Theses (TESEO) database Teseo (Ministry of Education and Science, Spain) was used in order to retrieve doctoral dissertations based on toxoplasmosis in Spain. This database compiles Spanish doctoral theses defended in Spanish universities since 1971 supported by the Spanish Ministry of Education. The protocol for this Meta-analysis was registered in Prospero (CRD42023411224). No language limitation was applied. The databases were searched using “toxoplasmosis”, “*Toxoplasma*”, “*Toxoplasma gondii*”, “prevalence”, “seroprevalence”, “HIV”, and “Spain'' as keywords. The combination of keywords and Booleans terms used for the research was as appears in annex I.

### Inclusion and Exclusion Criteria

After extracting the records from the databases, duplicate records and the titles and abstracts were screened for relevance. Original articles were set apart in two groups: (i) studies in humans and (ii) studies in animals. The human group was evaluated according to the following criteria: (1) Toxoplasmosis prevalence and seroprevalence, and (2) Studies conducted in humans in Spain. The exclusion criteria were as follows: (1) Toxoplasmosis in animals, (2) Reviews, (3) Toxoplasmosis studies with no prevalence data, (4) Studies where the total of the population was diagnosed with Toxoplasmosis, and (5) Toxoplasmosis studies in other countries than Spain. We excluded editorials, commentaries, letters to the editor, case–control studies and case series. After the screening process, full texts were evaluated using the same criteria described above.

### Study Selection and Data Extraction

Initial screening of manuscript titles and abstracts was performed independently by two researchers (MMV and PCN). It includes articles listed in Rayyan [19], software used to review collaborative and independent publications by different researchers. Articles were screened based on title, abstract and full text as well as doctoral dissertations listed in Teseo. Discrepancies were solved by discussion and agreement. If there were doubts or inconsistencies other researchers were consulted and a decision was made by consensus. The information extracted from each study includes author, period of study, year of publication, sample size, number of positive cases (IgG-anti *Toxoplasma* antibodies seropositive), *T. gondii* seropositivity rates (seroprevalence), diagnostic techniques and design type study. The articles were divided into three groups according to the target populations of this study: pregnant women and congenital cases, immunocompromised (HIV) patients and immunocompetent patients (any patient without pregnancy/congenital case). In addition, a separate study was also conducted for cerebral toxoplasmosis using data from HIV patients. (Table 1).

**Table 1:** Overview of the characteristics of the included studies

Targeted group	Years of study	Location	Age range	Seroprevalence (CI 95%)* n* IgG^+^/*N* total	Diagnostic technique	Design type study	References
Pregnant woman	2006–2010	Elche	16–45	12.0%^a^ (10,5–13.8) 179/1488 41.4%^c^ (38.9–43.9) 619/1495	ELISA	Cross-sectional	[21]
Pregnant woman	2007–2008	Granada	NR	17% (3.6–5.9) 602/3,541	ELISA	Transversal study	[22]
Pregnant woman	2001	Salamanca	21–38	18.8% (17.4–20.2) 552/2,929	EIA	Retrospective study	[23]
Pregnant women	1999	Barcelona	15–44	28.6% (28–29.3) 4,687/16,362	Serologic test	Prospective	[24]
Pregnant women	1993	Málaga	27.1^b^	25.7% (19.9–32.3) 49/191	Serologic test	Transversal	[25]
Pregnant women	1992–2008	Zaragoza	NR	31.9% (31.5–32.3) 15,207/47,635	ELISA	Retrospective	[26]
Pregnant women	2007–2010	Madrid	19–49	23.39% (22.5–24.3) 1,874/8,012 33,8%^c^ (32.0–35.6) 930/2752 18%^a^ (16.9–19.0) 946 /5,260	CLIA	Transversal	[27]
Pregnant women	2007–2008	Madrid	28.6^b^	30% (27.6–32.5) 427/1427 31%^c^ (28.6–33.6) 405/1,305 18%^a^ (12.2–25.8) 22/122	CLIA	Transversal	[28]
Pregnant women	1996–1997	Madrid	15–45	25.4% (24.5–26.3) 2,502/9,846	ELISA	Transversal	[29]
HIV Patient	1992–2009	Madrid	> 18	60.1% (54.5–65.0) 217/361	Serologic test	Transversal	[30]
HIV Patient	1997–1998	Madrid	24–63	16,98% (9.2–29.2) 9/53	NR	Retrospective	[31]
HIV Patient	1989–1997	Madrid	> 16	41.4% (38.4–44.5) 422/1115	Serologic test	Retrospective	[32]
Women^d^	1992–1999	Barcelona	15–45	40.7% (39.5–41.8) 2,883/7090	ELISA	Retrospective	[34]
Patients	2002–2003	Extremadura	2–93	36.1%^e^ (0.99–1.48) 644/1785 33.4%^f^ (30.2–36.5) 285/857	ELISA	Study	[35]
Children	year NR^g^	Madrid	2–14	42.8% (39.2–46.4) 311/727	DAT	Prospective	[36]
Pregnant women	2006	Albacete	30^b^	21% (19–22) 541/2,623 51% ^c^ (46–56) 930/2,752 16% ^a^ (14–17) 946 /5,260	EIA	Transversal	[37]
Hospital patients	1993–1996	Lerida	All ages	51.1% (49.2–53.0) 1,330/2,603	MEIA	Study	[38]
Hospital patients	NR	Gran Canaria	All ages	63.35% (58.6–67.8) 261/412	ELISA	Transversal	[39]

*n*: sample size, *IgG*^*+*^ IgG anti-*T.gondii* antibodies positive, *ELISA* Enzyme-Linked Immunosorbent Assay, *EIA* Indirect Enzyme ImmunoAssay, *CLIA* Chemiluminescent Immunoassay, *DAT* Direct Agglutination Test, *MEIA* microparticle enzyme immunoassay technology, *NR* not reported in the study, *CI* confidence Interval

^a^Spanish native pregnant women

^b^Average age of pregnant women analysed in the study

^c^Migrant pregnant women

^d^Women of childbearing age

^e^Rural

^f^Urban

^g^10 months of follow-up

### Assessment of Study Quality

Risk of bias was assessed using the Joanna Briggs Institute Critical Appraisal Tools checklist [20] for prevalence studies. This checklist includes items assessing the sample frame, recruitment of study participants, sample size, description of the study subjects and setting, coverage of the identified sample, methods for the identification condition, standardisation of the measurement of the condition, statistical analysis and the management for an adequate response regarding dropouts, refusals or “not found”, obtaining an overall appraisal as include, exclude or seeking for further information (Table 2).

**Table 2:** Critical appraisal checklist by both researchers

Reference	Was the sample frame appropriate to address the target population?		Were study participants sampled in an appropriate way?		Was the sample size adequate?		Were the study subjects and the setting described in detail?		Was the data analysis conducted with sufficient coverage of the identified sample?		Were valid methods used for the identification of the condition?		Was the condition measured in a standard, reliable way for all participants?		Was there appropriate statistical analysis?		Was the response rate adequate, and if not, was the low response rate managed appropriately?		Overall appraisal	
	Res1	Res2	Res1	Res2	Res1	Res2	Res1	Res2	Res1	Res2	Res1	Res2	Res1	Res2	Res1	Res2	Res1	Res2	Res1	Res2
[21]	Yes	Yes	Yes	Yes	Yes	Yes	Yes	Yes	Yes	Yes	Yes	Yes	Yes	Yes	Yes	Yes	Yes	Yes	1	1
[22]	Yes	Yes	Yes	Yes	Yes	Yes	Yes	Yes	Yes	Yes	Yes	Yes	Yes	Yes	Yes	Yes	Yes	Yes	1	1
[23]	Yes	Yes	Yes	Yes	Yes	Yes	Yes	Yes	Yes	Yes	Yes	Yes	Yes	Yes	Yes	Yes	Yes	Yes	1	1
[24]	Yes	Yes	Yes	Yes	Yes	Yes	Yes	Yes	Yes	Yes	Yes	Yes	No	No	Yes	Yes	Yes	Yes	1	1
[25]	Yes	Yes	Yes	Yes	Yes	Yes	Yes	Yes	Yes	Yes	Yes	Yes	Yes	Yes	Yes	No	Yes	Yes	1	1
[26]	Yes	Yes	Yes	Yes	Yes	Yes	Yes	Yes	Yes	Yes	Yes	Yes	No	No	Yes	Yes	Yes	Yes	1	1
[27]	Yes	Yes	Yes	Yes	Yes	Yes	Yes	Yes	Yes	Yes	Yes	Yes	No	No	Yes	No	Yes	Yes	1	1
[28]	Yes	Yes	Yes	Yes	Yes	Yes	Yes	Yes	Yes	Yes	Yes	Yes	Yes	Yes	Yes	No	Yes	Yes	1	1
[29]	Yes	Yes	Yes	Yes	Yes	Yes	Yes	Yes	Yes	Yes	Yes	Yes	Yes	Yes	Yes	Yes	Yes	Yes	1	1
[30]	Yes	Yes	Yes	Yes	Yes	Yes	Yes	Yes	Yes	Yes	Yes	Yes	Yes	Yes	Yes	Yes	Yes	Yes	1	1
[31]	Yes	Yes	Yes	Yes	Yes	Yes	Yes	Yes	Yes	Yes	Yes	No	Yes	Yes	Yes	Yes	Yes	Yes	1	1
[32]	Yes	Yes	Yes	Yes	Yes	Yes	Yes	Yes	Yes	Yes	Yes	Yes	Yes	Yes	Yes	Yes	Yes	Yes	1	1
[34]	Yes	Yes	Yes	Yes	Yes	Yes	Yes	Yes	Yes	Yes	Yes	Yes	Yes	Yes	Yes	No	Yes	Yes	1	1
[35]	Yes	Yes	Yes	Yes	Yes	Yes	Yes	Yes	Yes	Yes	Yes	Yes	Yes	Yes	Yes	No	Yes	Yes	1	1
[36]	Yes	Yes	Yes	Yes	Yes	Yes	Yes	Yes	Yes	Yes	Yes	Yes	Yes	Yes	Yes	Yes	Yes	Yes	1	1
[37]	Yes	Yes	Yes	Yes	Yes	Yes	Yes	Yes	Yes	Yes	Yes	Yes	Yes	Yes	Yes	No	Yes	Yes	1	1
[38]	Yes	Yes	Yes	Yes	Yes	Yes	Yes	Yes	Yes	Yes	Yes	Yes	No	No	Yes	Yes	Yes	Yes	1	1
[39]	Yes	Yes	Yes	Yes	Yes	Yes	Yes	Yes	Yes	Yes	Yes	Yes	Yes	Yes	Yes	Yes	Yes	Yes	1	1

Res1: Research 1

Res2: Research 2

Overall appraisal: Include (1)

### Statistical Analysis

Prevalence variance was determined for each study, and 95% confidence interval thereof. Prevalence rate from different studies was combined using weighted mean. The publication bias was examined using the Egger's test and funnel plot and the heterogeneity of the selected research works was evaluated using the *I*^2^ (%) test. An *I*^2^ > 75% was considered high heterogeneity and a random-effects model was employed for the analysis. Pooled prevalence was calculated as an event rate. The association between the prevalence of toxoplasmosis and the middle year of sampling of the seroprevalence study (“midyear”) was examined using meta-regression analysis. Subgroup analysis was performed by midyear and by location for Madrid area and Catalonia-Andorra area. The Comprehensive Meta-Analysis (Version 2) (Biostat, Englewood, NJ, USA) software was used to analyse the data.

## Results

**Fig. 1:**
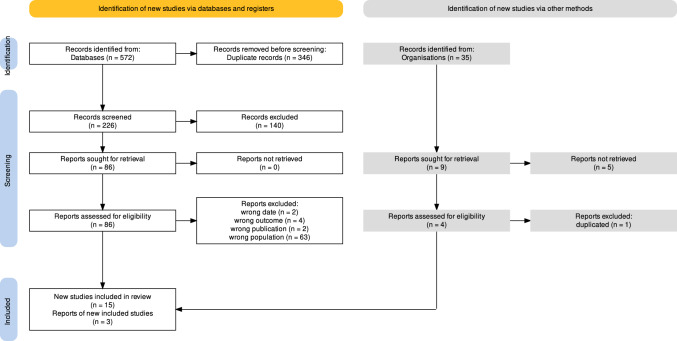
Flow diagram of the study design process

Overall, 572 studies were identified along with 35 records from doctoral thesis. A total of 346 records were excluded based on the inclusion/exclusion criteria and 15 studies along with three doctoral theses were eligible for inclusion in this systematic review and meta-analysis (Fig. 1). Eligibility was performed individually by each reviewer with a 0.901 Cohen’s Kappa of coincidence. Quality assessment was also separately done with a 0.613 Cohen's Kappa coefficient of agreement. Among the included articles in this systematic review, the most common diagnostic method used was enzyme-linked immunosorbent assay (ELISA) in eight studies (8/18, representing a 44.4% of the studies), followed by chemiluminescence immunoassay (CLIA 2/18, representing 11.1% of the studies) and direct agglutination test (DAT 1/18; 5.56%) (Table 1).

Ten studies focused on pregnant women and congenital cases, nine articles [21–28] and one doctoral thesis [29] were analysed. A total of 24,737 seropositive cases were found with a rate 24,737/85,703. Age range for this targeted group was 15–45 years old. A wide variety of areas was found covering data for Barcelona, Granada, Salamanca, Elche, Madrid, Malaga and Zaragoza. In the majority of the reports (4/10, 40%) was found that it was used ELISA to detect *T. gondii* antibodies in human sera followed by CLIA (2/10, 20%).

Regarding immunocompromised population, three studies were found [30–32] which included 1,529 sera samples from HIV patients. A total of 648 samples were found positive to *Toxoplasma gondii* antibodies. This group age range was older than 16 years old. Sample data was extracted mainly in Madrid. In order to detect *T. gondii* antibodies ELISA method was mainly used although DAT was found to be used in one of the reports for this group (33.3%). In addition, data regarding cerebral toxoplasmosis in AIDS patients were obtained from three studies [30, 32, 33], and the ratio seropositive case/total was 2430/23725.

Four studies on immunocompetent patients were analysed [34–37] and two doctoral theses [37, 38] which included 23,338 sera samples from women in childbearing age and people between 2 and 93 years old and hospitalised patients were assessed. A total of 8222 samples were found seropositive (8222/23,338). Sample data was extracted in Barcelona, Extremadura, Lerida and Gran Canaria Island. In order to detect *T. gondii* antibodies an ELISA method was used in most of the studies (3/4, 75%) although MEIA methods were found to be used in the fourth report.

### Meta-Analysis

**Fig. 2:**
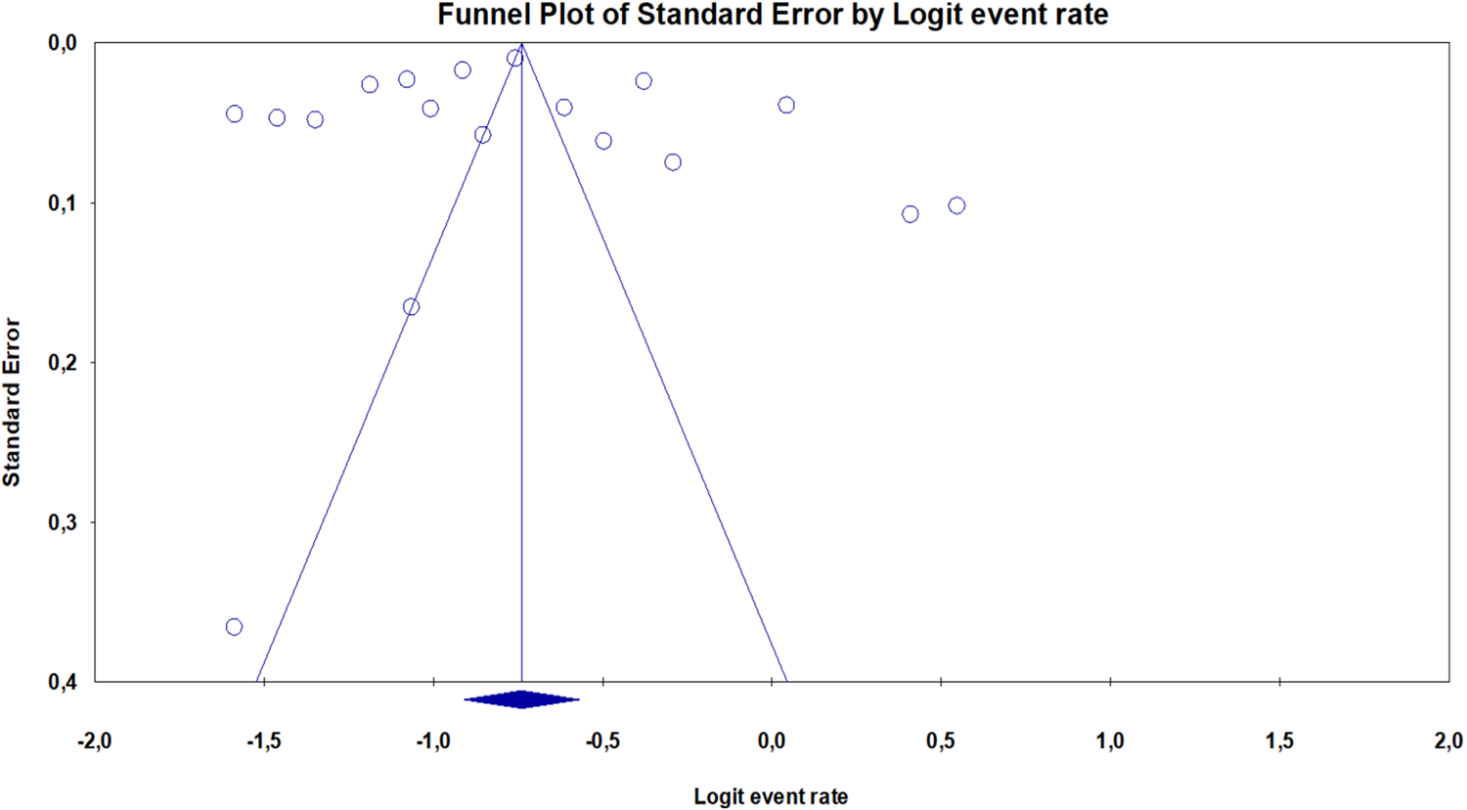
Funnel Plot Results of the prevalence of toxoplasmosis in Spanish population

**Fig. 3:**
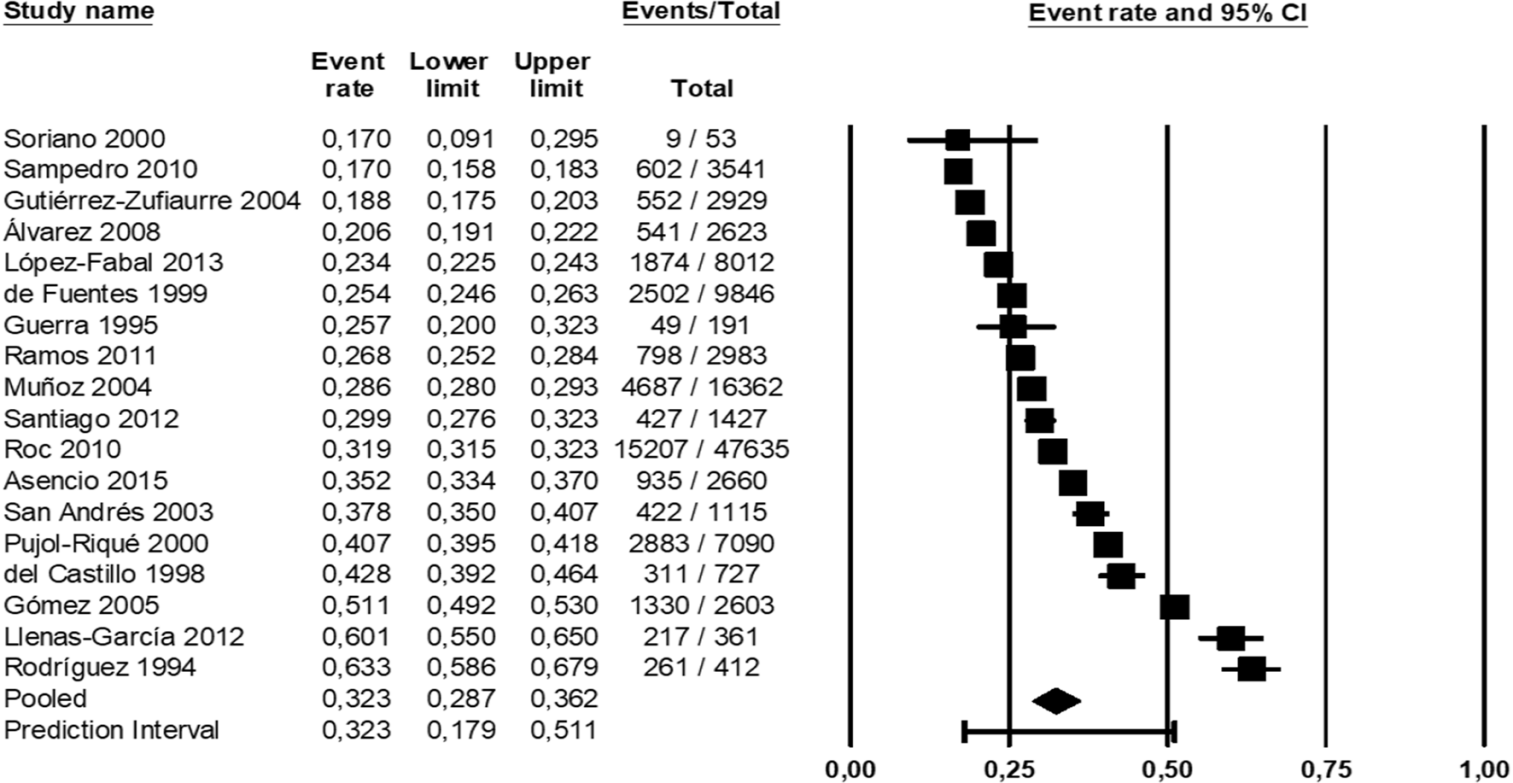
Forest plot of toxoplasmosis seroprevalence in Spanish population with a 95% confidence interval based on random effect model

A random effects model was used to integrate the studies and share the prevalence estimate because of the heterogeneity of the studies that were chosen and the test findings (*I*^*2*^: 99.97%). With a significance level of 0.05, the probability of publication bias in the findings of the prevalence of toxoplasmosis in the Spanish population by funnel diagram and Egger's test (Fig. 2) revealed no publication bias of the prevalence in the current study (*P* = 0.595). In an investigation of 18 studies with a total sample size of 110,570 people in the age range between 2 and 93 years old, a total of 33,607 positive cases were found. The prevalence of toxoplasmosis in the overall Spanish population was 32.3% (95% CI 28.7–36.2%), according to the findings of the study in the forest plot (Fig. 3).

**Fig. 4:**
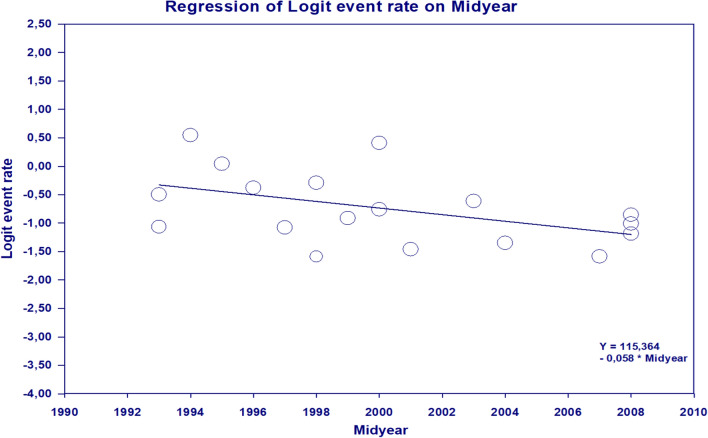
Meta-regression chart of the prevalence of toxoplasmosis in Spanish population by middle year of study

**Fig. 5:**
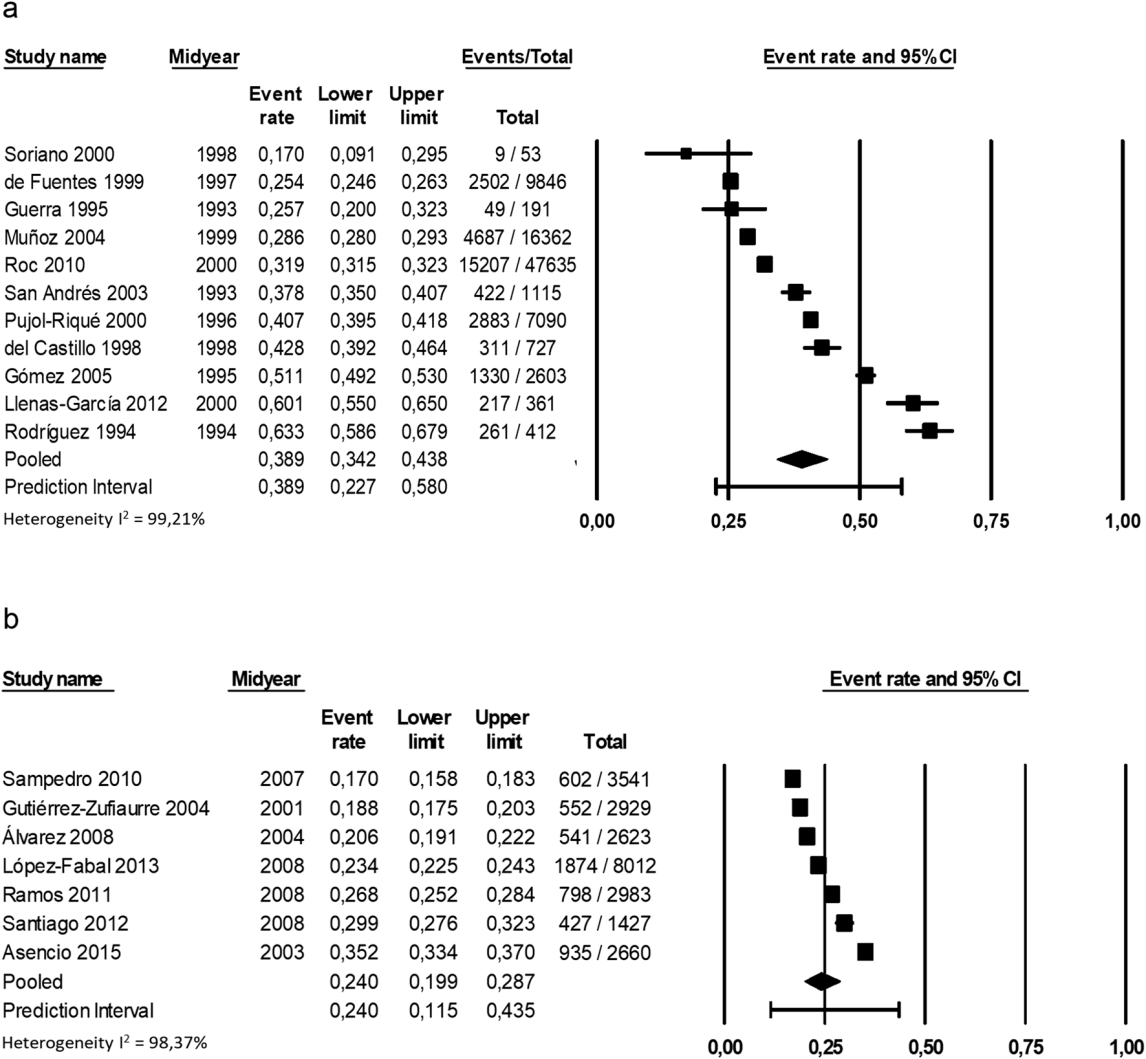
Forests plots of toxoplasmosis seroprevalence in Spanish population according to temporality. **a** Studies with data from the twentieth century and **b** from the twenty-first century

**Fig. 6:**
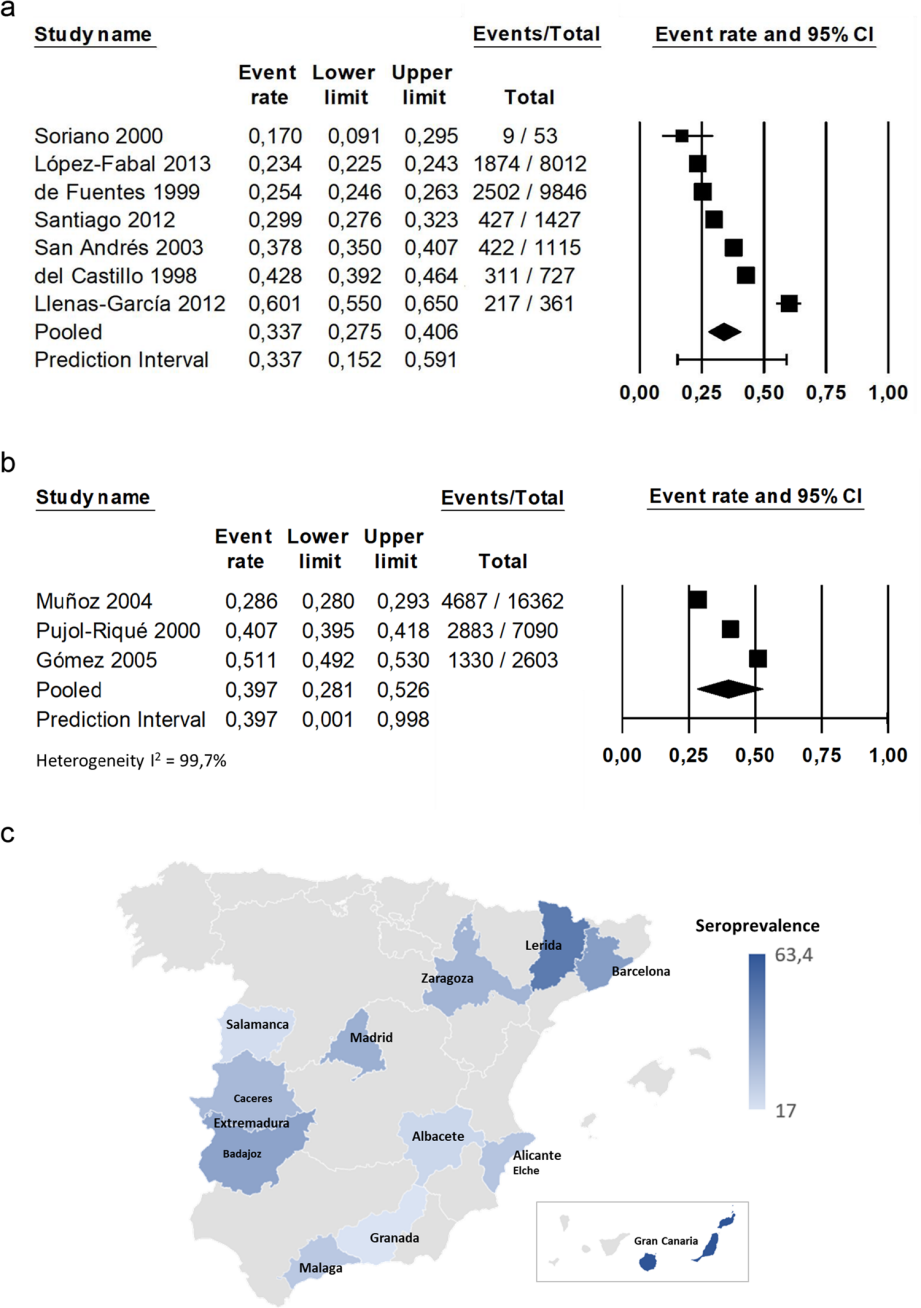
Forests plots and geographical representation of toxoplasmosis seroprevalence in Spanish population regarding location. **a** Studies with data from the Community of Madrid; **b** studies with data from Catalonia and Andorra; **c** map of Spain with the distribution by provinces of the seroprevalence studies included in the review

Our meta-regression results showed that the prevalence of toxoplasmosis decreased in the Spanish population with increasing sampling years, being the difference over decades statistically significant (*P* = 0.0255) (Fig. 4); this value indicates that the decreased in seroprevalence rate for toxoplasmosis throughout the 3 decades studied was significant, being *P* = 0.0255 less than 0.05. These results were consistent with the analysis by subgroups performed using “midyear” moderator, were samples from twentieth century (1993–2000) showed a pooled prevalence of 38.9% (95% CI 34.2–43.8%) (Fig. 5a) whereas samples from twenty-first century (2001–2008) presented a pooled prevalence of 24.0% (95% CI 19.9–28.7%) (Fig. 5b), with a reduction of a 14.9%. I^2^ for twentieth and twenty-first century studies were 99.21 and 98.37 respectively and Egger’s test detected no bias (*P* = 0.175, *P* = 0.983). Analysis by location (Fig. 6c) showed a prevalence of 33.7% (95% CI 27.5–40.6%) for Madrid area (Fig. 6a) and 39.7% (95% CI 28.1–52.6) for Catalonia-Andorra area (Fig. 6b), with an I2 of 98.49% and 99.7% respectively and without significant publication bias according to Egger’s test (*P* = 0.080, *P* = 0.198).

**Fig. 7:**
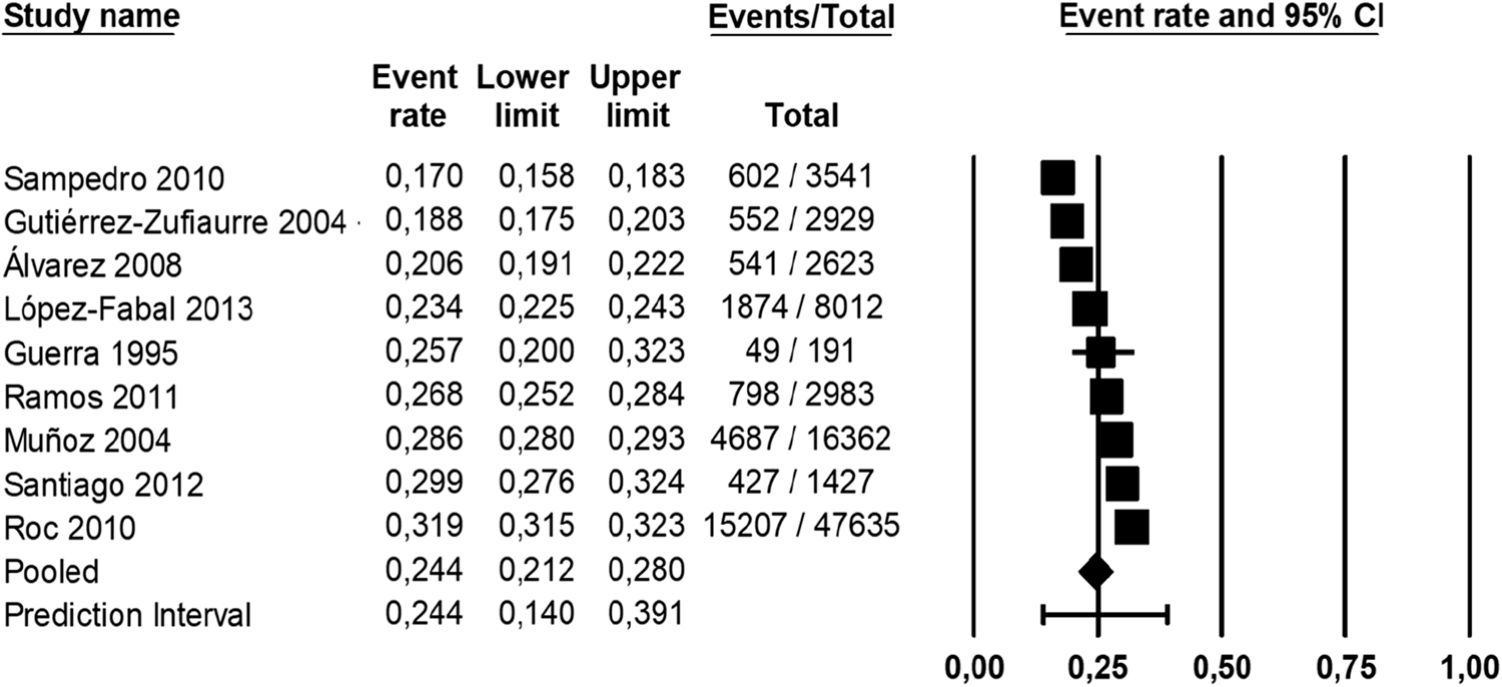
Forest plot of toxoplasmosis seroprevalence in pregnant women in Spain with a 95% confidence interval based on random effect model

The meta-analysis for the pregnant women (Fig. 7) reported that the pooled prevalence of toxoplasmosis in pregnant women in Spain was 24.4% (24,737/85,703, 95% CI 21.2–28.0%), based on the random effects model. Heterogeneity among prevalence in pregnant women was evaluated (*I*^*2*^: 99.06%) and Egger’s test results for publication bias at a significance level of 0.05 revealed that there was no publication bias in the current study (*P* = 0.259).

**Fig. 8:**
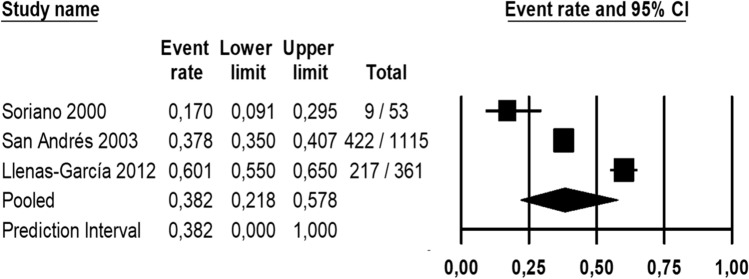
Forest plot of toxoplasmosis seroprevalence in immunocompromised population in Spain with a 95% confidence interval based on random effect model

**Fig. 9:**
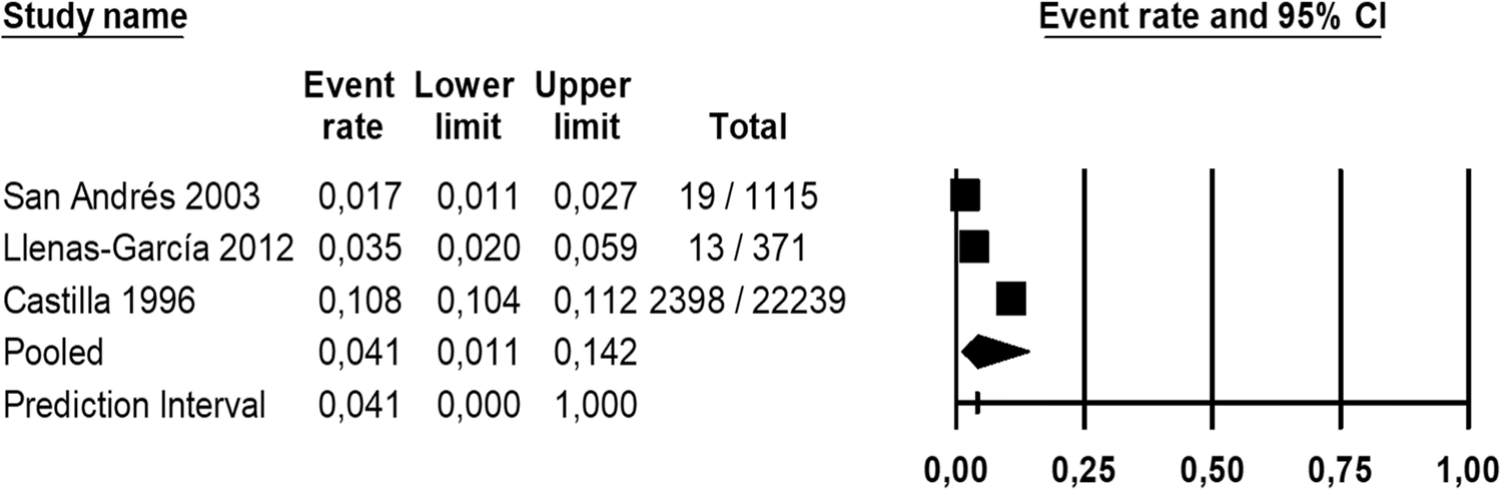
Forest plot of cerebral toxoplasma in immunodeficient population in Spain with a 95% confidence interval based on random effect model

The meta-analysis for immunocompromised population (Fig. 8) reported that the pooled seroprevalence of toxoplasmosis among the immunodeficient in Spain was 38.2% (95% CI 21.8–57.8%), based on the random effects model. Heterogeneity among seroprevalence in the immunodeficient population was calculated (*I*^*2*^: 97.06) and Egger’s test results for publication bias at a significance level of 0.05 revealed that there was no publication bias in the current study (*P* = 0.194). Pooled incidence for cerebral toxoplasma among HIV patients was also calculated (Fig. 9) being in Spain 4.1% (95% CI 1.1%-14.2%), based on the random effects model. Heterogeneity among cerebral toxoplasma incidence in the immunodeficient population was calculated (*I*^*2*^: 98.34%) and Egger’s test results for publication bias at a significance level of 0.05 revealed that there was no publication bias in the current study (*P* = 0.207).

## Discussion

*Toxoplasma gondii* infections are common worldwide, and many of them are asymptomatic. However, *T. gondii* can cause severe disease in humans, especially in congenitally infected children and the immunocompromised population. It is reported that toxoplasmosis causes 24% of the hospitalizations linked to a foodborne illness in the USA and it might lead to death even for the immunocompetent population [40].

It is considered essential to have knowledge about the epidemiological data regarding prevalence of toxoplasmosis worldwide and, specifically in Spain, considering, among other aspects, the importance of the use of gestational screening in the diagnosis of congenital toxoplasmosis [41]. Identifying the evolution and current situation of prevalence can contribute to a better understanding of the epidemiology of *T. gondii* in the country, to know the risk and rate of exposure to the parasite, among other factors. In this way, health professionals can be supported to control this disease and avoid its consequences. It can help health authorities establish appropriate measures to address this serious public health problem and would be useful in the formulation of improved public health policies such as the implementation of prenatal detection and treatment programs.

Toxoplasmosis prevalence among the different geographical regions might differ due to different conditions such as the weather, lifestyles or economic factors [42, 43]. There are different factors that explain the variations in the prevalence of the disease within different areas of the same country, such as climatic and environmental differences, depending on whether they are rural or urban areas, the possibility of greater contact with stray cats, social conditions with habits such as the consumption of raw or semi-cooked meat, among others. Different environmental conditions and climatic parameters, such as annual rainfall, can influence the greater or lesser prevalence of toxoplasmosis, with studies observing that precipitation, humidity and temperature influence the survival and infectivity of *T. gondii* oocysts in the environment [44]. For this reason, being aware of the seroprevalence data in Spain can lead to design and implement guidelines in the prevention and control of the disease, including the follow-up protocol for immunocompromised patients or the screening in pregnant women to avoid the risk of vertical infections. It is known that the implementation of these guidelines leads to a decrease in the seroprevalence ratio, although in countries like Spain it is not a common practice implemented by law [41]. Authors suggest that transmission routes vary depending on period of time, in the 90 s the ingestion of meat with *T. gondii* cysts was considered the main source of infection, in the 2000s oocysts in water, soil, fruits and raw vegetables were considered of great importance, as main spreading pathways of the disease [45].

National seroprevalence data in Spain is limited and their evolution over the years is unknown. Furthermore, it is interesting to identify the prevalence in the main identified risk groups: pregnant women, immunocompromised (HIV patients or transplant recipients), and immunocompetent population. This investigation tries to compile and comprehensively assess all the available information for seroprevalence in Spain during the last three decades.

### Seroprevalence of Toxoplasmosis in the Spanish Population

This systematic review was conducted following the Preferred Reporting Items for Systematic Reviews and Meta-analysis (PRISMA) and its checklist [18]. Four scientific databases (Web of Science, PubMed, Scopus and Teseo) were searched for studies related to human toxoplasmosis published up to December 2023 in Spain. The pooled seroprevalence in Spain between 1993 and 2023 calculated in this review was 32.3% (95% CI 28.7–36.2%). This seroprevalence is lower than other regions in Europe such as France (54.7% [46]), Romania (41%) [47], Germany (63%) ([48, 49]) or Ireland (34%) [48], although it was reported a seroprevalence lower than in Spain in countries like Portugal (24%) or Greece (25%) between 2004 and 2008 [48]. Compared with other non-European regions, the data obtained in this study is higher than in the USA and China, with a seroprevalence of 11%, but lower than in South America (Mexico 49%, Peru 39% and Brazil 50%) or Africa (Cameroon 77% and Ethiopia 74%) [48]. It is known that many aspects could modify the seroprevalence of toxoplasmosis such as hygienic and dietary habits, climate conditions, socioeconomic factors, contact with definitive hosts (felids), among others; all these points can be used in order to explain the difference between seroprevalence data worldwide [4]. Directly related with the human seroprevalence for toxoplasmosis is the data for animals, especially for cats, since they are a main actor of transmission for the disease. In a study published in 2004 from Spain, the overall prevalence observed for this group of animals was 25.5% in household cats and 36.4% in stray groups [50]. This result was observed to be lower in a study-conducted years later; the seroprevalence of *T. gondii* infection was 24.2% for free-roaming cats living in urban areas [51]. However, in a recent study in wild felids, a seroprevalence of 45% in lynx and 85% wild cat was found, showing a wide dissemination of the parasite in the wild environment [52]. Being aware of the role of the felids in the epidemiology of *T. gondii* is important since each infected felid can shed millions of oocysts that can spread the infection to many other susceptible hosts [53].

In our study a decreasing trend throughout the last three decades in seroprevalence was found in Spain for humans assignable to many factors such as improved sanitising methods for cats owners and a lower risk in domestic cats (mostly fed with feed), have made it possible to reduce the burden of disease in different territories, or better food habits from consuming raw or contaminated meat, especially due to the most commonly widespread habit of freezing meat before consumption, which causes the bradyzoites found in parasitized meat to die, avoiding infection of the consumer. or the implementation of sanitising measures such as ionising radiation in [54, 55].

This decline situation can be supported by the analysis of the number of hospitalizations in Spain due to toxoplasmosis. Although it is evident and well known that the majority of cases of toxoplasmosis do not require hospitalisation, knowing the recorded cases of hospitalisation for this cause can be an approach to understand the situation, since we do not have complete and reliable records on the true burden of disease in our environment. According to Estevez Reboredo et al. (2021) [56], in a study published using the interactive platform RAE-CMBD, specialised health care registry of the National Health System, Spanish Ministry of Health (Registro de Actividad de Atención Especializada. RAE-CMBD, Ministerio de Sanidad) from the minimum basic dataset of the Spanish national registry of hospital discharges and based on the number of hospitalizations linked to toxoplasmosis diagnosis. The study focused on hospital discharges with a diagnosis of “toxoplasmosis” during the period 1997–2018. Frequencies and rates of hospital discharges per 100,000 inhabitants (TH) were carried out according to sex and age groups, finding a remarkable decline in the number of patients discharged with a toxoplasmosis diagnosis (TH = 1,55 in 2000 to TH = 0,48 in 2021), with a significantly greater decrease in men than in women. The highest hospitalisation rate corresponded to the age group 15–44 years. Another study completing the previously indicated one, also using the RAE-MBD platform analysing hospital discharges with a diagnosis of “toxoplasmosis” during the period 2000–2021, coincided in the observation of the progressive decrease in both in the number of hospitalizations and annual TH, being significantly higher in men than in women and those most affected were middle age groups [57]. These studies may show a lower incidence of the disease, at least a clear decrease in records of serious cases requiring hospitalisation. The greatest progressive decrease was in men, mainly in the age group of 30 to 44 years, which could be related to the implementation of highly active antiretroviral treatment (HAART) in HIV-positive patients, which contributes to the reduction of concomitant processes by opportunistic agents such as *Toxoplasma i*n immunosuppressed patients [32]. This, together with the factors of hygienic measures and changes in consumption habits and contact with animals, previously indicated, support the trend of decreasing prevalence of this disease.

However, it should be noted that although a decrease in prevalence has been identified, it is still notable and practices such as organic farming with natural fertilisers and without treatments, some culinary and cultural practices and the maintenance of colonies of stray cats among other risk factors, put the risk of contracting the disease, especially in the more susceptible and immunocompromised.

Despite the remarkable degree of heterogeneity observed, the frequency of exposure to *T. gondii* is high and widespread. In our research, the toxoplasmosis data was extracted from studies carried out in various areas or provinces from Spain (Zaragoza, Salamanca, Barcelona, Lerida, Madrid, Elche, Granada, Malaga, Extremadura and Gran Canaria Island). Spain, located on the western peninsula of the European continent, is characterised by having high climatic diversity and a territory with great biodiversity and the presence of wild animals that can play an important role in the epidemiology of pathogens with relevance to public health. [58]. The northern and eastern areas of the peninsula and islands are characterised by mild temperatures and a higher average annual rainfall, with humidity that allows the survival of oocysts in the soil, plants and water for several years, being a potential source of infection for humans and animals [53]. The central and interior area of the Iberian Peninsula is a region where temperature changes are more pronounced, with cold winters, hot and dry summers and less rain, being less appropriate for the survival of oocysts.

This review highlights a widespread exposure to *Toxoplasma* in Spaniards, but appears to have higher rates of anti-*T. gondii* antibodies in northern Spain and island, as in the studies in Barcelona [24, 34], Lerida [38] or the Gran Canaria Island [39] compared to those reported in the peninsula hinterland as the studies in Granada [22]; Salamanca [23] or Albacete [37]. This finding may be related to the geographical and climatic differences observed, since in animal studies a significantly higher seroprevalence of *T. gondii* has been reported in wildlife animals from wetter areas of northeaster Spain than in those from central and southern regions of the country [59]. The geographical differences in the observed seroprevalences could be related to the type of habitat, greater or lesser presence of felids or infected animals, and environmental factors that may influence the persistence of viable oocysts contaminating the environment.

Different seroprevalence rates are found during the last three decades ranging 12% (Elche [21]) -63.3% (Gran Canaria Island [39]). A wide rate of seroprevalence is observed if comparing the same areas' seroprevalence rates, such as Madrid (17%- 60.1%), or Barcelona (28.6%-40.7%). Madrid, located in the centre of the country, is the area in which the most studies have been included in the meta-analysis. A prospective study in children (2–14 years) published in 1998 showed a higher seroprevalence (42.8%) reflecting the higher seroprevalence found in the early 1990s [36].Those carried out on women of childbearing age during routine pregnancy screening, which more closely reflect the general population, showed average seroprevalences of 25.4% [29], 29.9% (although in this study differentiated the seroprevalence of women born in Spain (18%) versus migrants (31%) [28] and 23% [27], observing a certain decrease in relation to the time period analysed. Studies in HIV positive patients in Madrid showed very variable seroprevalences varying from 17% [31] to 60% [30], influenced by the heterogeneity of the studies.

The higher seroprevalence rate is detected in Las Palmas de Gran Canaria (63.35%) and Elche with 12%, the lower outcome. Seroprevalence data has been obtained during thirty years so, it is observed that for the 90 decade the outcome was higher (Las Palmas de Gran Canaria, 63.35%, Madrid, 60.1%- 41.4%) and it has been decreased with the decades (Madrid with 18% between 2007 and 2010).

### Seroprevalence in Pregnant Women

*Toxoplasma gondii* infection in pregnant women occurs worldwide with frequencies between 0.1 and 1% and approximately 40% of unborn children are infected [60, 61]; on the other hand, many infected children are asymptomatic at birth but suffer sequelae in long-term life [60–62]. In France, a universal antenatal screening is recommended as a strategy to decrease the number of congenital cases and vertical transmissions [63]. It is stated by the researchers of a study published in France that the policy of screening during pregnancy would avoid the nation €148 million thus reducing or eliminating sequelae [64]; however, there is still a controversy regarding the screening programs and, certain countries like Spain or Switzerland, have stopped it despite the beneficial outcomes of the programs [65]. Moreover, in a report by ECDC (European Centre for Disease Control) [14] published in 2020 and focused on congenital toxoplasmosis, it is stated that, although the number of cases has decreased between 2016 and 2020 in the European Union, the burden of congenital toxoplasmosis cannot be assessed due to many differences in national surveillance systems and screening programs. In a study focused on global, regional and country seroprevalence in pregnant women conducted in 2018, researchers stated that the global seroprevalence was 32.9%, finding the seroprevalence in the Americas as high as 45.2% and the lowest data for Western Pacific with 11.2% [66]. In Europe a global seroprevalence of 31.2% (28.4– 34.0%) [67] was found but differing in different countries such as in Italy, where it was observed the global prevalence in pregnant women was estimated in a decreasing trend between 27.5% to 21.5% [68, 69]. In Spain, the global prevalence of latent toxoplasmosis for pregnant women was estimated at 33.8% [67]. According to our study, the pooled seroprevalence in Spain for this targeted group was 24.4% (95% CI 21.2–28.0%) which includes 85,703 samples from pregnant women and congenital cases, and a total of 24,737 seropositive cases, this value is slightly lower than the seroprevalence obtained in other countries of Europe, such as France, where the seroprevalence found in a study published in France surveying national perinatal data was 31% [70]. When compared to other regions of the world, the overall seroprevalence of *Toxoplasma gondii* in pregnant women in Spain shows an intermediate seroprevalence between the highest value for the Americas (45.2%, 95%CI 33.4–53.4) and the lowest data found for the Western Pacific (11.2%, 7.8–15.1) [66]. However, in our review, many studies comparing seroprevalence on foreign patients and Spaniards were analysed and the result for the foreigners was higher than that of pregnant women born in Spain [21, 22, 27, 28]. According to these results, it can be stated that Spanish women have experienced a rise in toxoplasmosis antibodies in the last decades compared with patients from other continents such as Central or South America, who show a higher seroprevalence (37.5%, [28]). In general, the higher seroprevalence is found in patients with low-income countries, as one study states [66] or the contact with pets, mainly cats [28].

### Seroprevalence in Immunocompromised

In immunocompromised patients, such as HIV-infected patients and organ transplant recipients, the disease can affect the central nervous system, but can also affect any organ [71]. In this review, three studies prior to 2009 were analysed regarding this targeted group in Spain. No further data for the last decade was found, possibly due to a reduction in the number of HIV positive cases notified in the last 3 decades. The last updated data (June 30–2023, [72]) shows 331 diagnosed cases of HIV in Spain; this is a lower number compared with 1,511 diagnosed cases in 2003 [72]. In a recent review studying the global status for the seroprevalence of *T. gondii*, the pooled prevalence of this disease among the living population suffering from HIV was 44.22% [73]; this information is supported with similar findings in a study focused on the survival for HIV patients in Spain. In this study, the researchers state that the number of patients who survive increased from 1987 with the use of cART (combination antiretroviral treatment) treatments [74]. This statement is supported by the results in a retrospective analysis of 472,269 patients infected with HIV in Spain. The study used population-based data extracted from the minimum basic dataset of the Spanish National Registry of Hospital Discharges, 2020 (Registro de Atención Sanitaria Especializada, RAE-CMBD, Ministerio de Sanidad); the authors found that 9006 people presented *Toxoplasma gondii* infection [75]. According to this study, infection declined between 1997 and 2015, possibly due to the development of cART. In this review, a seroprevalence of 38.2% (95% CI 21.8–57.8%) was reported for immunocompromised patients from Spain, overall HIV population. This seroprevalence rate is slightly higher than the data reported in the UK which was 27% in 1990 [76] although it is lower compared with the prevalence rate found in Brazil, 72.9% [77] or India with 73% [78]. Some factors, as mentioned above, must be considered in order to justify these differences among seroprevalence rates, such as socioeconomic status in the population, climate conditions or sanitising habits.

For HIV patients, whose immune system is compromised, the infection caused by bradyzoite can be reactivated transformed into cytotoxic tachyzoites, causing cerebral mass lesions [79]. Cerebral Toxoplasmosis occurs as a result of a reactivation of the disease rather than a new infection when a patient is diagnosed with HIV [79]. In a study conducted in the USA, 79% of HIV patients developed cerebral toxoplasmosis [73]. In the present review, a meta-analysis was conducted using data for cerebral toxoplasmosis obtained from HIV patients, observing an incidence for this group of 4.1% (95% CI 1.1%-14.2%). According to Lau et al. (2021) [80], in a high-income country like Spain, the use of (cART) could lead to a decrease in the cerebral toxoplasmosis disease.

*Toxoplasma gondii* can also affect other types of immunocompromised patients such as, for example, organ transplants patients or cancer cases. Some studies are found in regards to the seroprevalence for *T. gondii* in cancer patients, showing a worldwide seroprevalence of 30.8% [81] and 20% in Egypt [82]; however, no data was obtained in this study for results of seroprevalence in cancer patients in Spain.

Although this study has found a decrease in the seroprevalence of Toxoplasmosis in Spain throughout the three last decades confirmed with a *P* = 0.0255 (less than 0.05 to be significant), researchers state that a lack of more data and studies was detected. It is considered fundamental to be tenacious on more investigations on Toxoplasmosis prevalence. It is necessary to obtain information about the prevalence rate of Toxoplasmosis in Spain in order to design actuations which help to reduce the infection and design educational programs to minimise its consequences. Furthermore, it is necessary to consider that Toxoplasmosis is an under-reported disease in Spain [83] so it is difficult to have accurate data for some of the targeted groups such as cancer or transplanted patients.

Our study has two main strengths. On the one hand, it is the first study in Spain dedicated to compiling and analysing previously published human seroprevalence data. On the other hand, the scope of this study focuses on a wide range of years (from 1993 to 2023). In addition, a high quality assessment (Johanna Briggs' Critical Appraisal Checklist for studies reporting prevalence data) was included. Secondly, this review includes not only human seroprevalence data, but also prevalence results for cerebral toxoplasmosis, which is associated with AIDS and disease reactivation. In relation of the limitations of the study, we highlight the lack of data for the years of the last decade (from 2015 onwards) in Spain. This hampers the comparison of data between decades and evidences the gap in the number of studies on human toxoplasmosis. In addition, the available seroprevalence data are not representative of all regions of the country, which hinders the comparison.

Regarding to the meta-analysis, the heterogeneity among studies was substantial. The *I*^*2*^ statistic is 99,97%, which tells us that some 99,97% of the variance in observed effects reflects variance in true effects rather than sampling error. Differences in sample size, sampling error, study year, or study province can all be contributing factors to the heterogeneity between studies, although heterogeneity has been reported to be often high among prevalence meta-analysis [84]. However, with an *I*^*2*^ > 75%, a study of moderators should be done, such as the location of the study and the year of sampling. Locations from the studies were difficult to group, since there was an enormous variation and only prevalence for Madrid area and the Catalonia-Andorra area could be calculated (Fig. 6). The rest of locations presented an *n* = 1, which made statistical analysis impossible. Madrid area had bigger representation (7 studies) and showed a very similar prevalence to overall Spanish prevalence (33.7% and 32.3% respectively). However, prevalence for Catalonia-Andorra area was higher (39.7%). This could mean that location is affecting heterogeneity, although low number of studies were found (*n* = 3). In order to evaluate changes across the decades and the possibility for this cause of heterogeneity among the studies, the prevalence of toxoplasmosis was analysed using a meta-regression model based on the middle year of the recruitment period of the sample of each study, which confirmed that the prevalence was being affected by temporality (*P* = 0.0255) (Fig. 4). This influence was also assessed analysing subgroups by the midyear moderator, with a 14.9% decrease in prevalence from one decade to another (Fig. 5), thus being a likely cause of heterogeneity in prevalence among studies.

## Conclusions

To the best of our knowledge, this is the first review and meta-analysis focused on the study of the toxoplasmosis seroprevalence in Spain that provides data for the last three decades (1993–2023). A decrease in the seroprevalence of toxoplasmosis has been observed throughout the period studied. The estimated pooled seroprevalence is 28.4% (95% CI 14.4–44.9%) slightly lower than in Europe. However, the lack of updated data in the general population and especially in some target groups, gives us a reason to demand more research on this topic. Furthermore, more initiatives in educational programs and appropriate control measures would help reduce this prevalence and better understand the disease.

## Supplementary Information

Below is the link to the electronic supplementary material.

Supplementary file1 (DOCX 13 KB)

## Data Availability

Data is contained within the article or supplementary material.
