# Supplementary material for: Prevalence of Human Toxoplasmosis in Spain Throughout the Three Last Decades (1993–2023): A Systematic Review and Meta-analysis
Source: J Epidemiol Glob Health. 2024 Jun 12;14(3):621–37. doi: 10.1007/s44197-024-00258-w (PMC11444046; doi:10.1007/s44197-024-00258-w)
Supplement: Supplementary file 1 — Supplementary file1 (DOCX 13 KB) [file 44197_2024_258_MOESM1_ESM.docx]

Search routes

***PubMed***

[*https://www.ncbi.nlm.nih.gov/pmc/articles/PMC6682582/*](https://www.ncbi.nlm.nih.gov/pmc/articles/PMC6682582/)

*("toxoplasmosis"[MeSH Terms] OR "toxoplasma"[MeSH Terms]) AND ("prevalence"[MeSH Terms] OR "seroepidemiologic studies"[MeSH Terms]) AND "spain"[MeSH Terms]*

***WoS***

***(TS=("Toxoplasmosis") OR TS=(toxoplasma gondii)) AND (TS=(seroprevalence) OR TS=(prevalence))AND TS=(spain)***

***137 results-con filtro 1993-2023 )***

1. DATABASE SEARCH STRATEGIES

**1.1 Pubmed**

Date of search: 21/02/2023

Search:

("Toxoplasmosis/epidemiology"[Mesh]) AND "Spain"[Mesh]

Filter: “Publication Years: 1994-2023”.

Results: 69

Date of search: 21/02/2023

Search:

("toxoplasmosis"[MeSH Terms] OR "toxoplasmosis"[All Fields] OR "toxoplasmosis"[All Fields]) AND ("seroepidemiologic studies"[MeSH Terms] OR ("seroepidemiologic"[All Fields] AND "studies"[All Fields]) OR "seroepidemiologic studies"[All Fields] OR "seroprevalence"[All Fields] OR "seroprevalences"[All Fields] OR "seroprevalance"[All Fields] OR "seroprevalances"[All Fields] OR "seroprevalency"[All Fields] OR "seroprevalent"[All Fields]) AND "spain"[MeSH Terms]

Results: 58

Date of search: 20/04/2023

Search:

("toxoplasmosis"[MeSH Terms] OR "toxoplasma"[MeSH Terms]) AND ("prevalence"[MeSH Terms] OR "seroepidemiologic studies"[MeSH Terms]) AND "spain"[MeSH Terms]

Results: 60

Search:

("toxoplasmosis"[MeSH Terms] OR "toxoplasmosis"[All Fields] OR "toxoplasmosis"[All Fields]) AND ("hiv"[MeSH Terms] OR "hiv"[All Fields]) AND ("epidemiology"[MeSH Subheading] OR "epidemiology"[All Fields] OR "prevalence"[All Fields] OR "prevalence"[MeSH Terms] OR "prevalance"[All Fields] OR "prevalences"[All Fields] OR "prevalence s"[All Fields] OR "prevalent"[All Fields] OR "prevalently"[All Fields] OR "prevalents"[All Fields]) AND ("spain"[MeSH Terms] OR "spain"[All Fields] OR "spain s"[All Fields])

Results: 30

1.1.1. Update Search

Date of search: 04/07/2023

Search:

("toxoplasmosis"[MeSH Terms] OR "toxoplasmosis"[All Fields] OR "toxoplasmoses"[All Fields]) AND ("seroepidemiologic studies"[MeSH Terms] OR ("seroepidemiologic"[All Fields] AND "studies"[All Fields]) OR "seroepidemiologic studies"[All Fields] OR "seroprevalence"[All Fields] OR "seroprevalences"[All Fields] OR "seroprevalance"[All Fields] OR "seroprevalances"[All Fields] OR "seroprevalency"[All Fields] OR "seroprevalent"[All Fields]) AND "spain"[MeSH Terms]

Results: 58

**1.2 Web of Science**

Date of search: 20/04/2023

Search:

Toxoplasmosis (Topic) and Seroprevalence (Topic) and Spain (Topic).

Results: 51

Date of search: 20/04/2023

Search:

Toxoplasmosis (Topic) and Epidemiology (Topic) and Spain (Topic).

Results: 34

Date of search: 25/04/2023

Search:

(TS=("Toxoplasmosis") OR TS=(toxoplasma gondii)) AND (TS=(seroprevalence) OR TS=(prevalence))AND TS=(spain)

Filter: “Publication Years: 1994-2023”.

Results: 134

23/10/23

<https://pubmed.ncbi.nlm.nih.gov/?term=toxoplasmosis%20hiv%20prevalence%20spain&sort=date&page=3>

toxoplasmosis hiv prevalence spain

30 results

30/10/23

<https://pubmed.ncbi.nlm.nih.gov/?term=hiv+toxoplasma+spain+prevalence&sort=date>

toxoplasma hiv prevalence spain

14 results

Update 13/2/24

<https://pubmed.ncbi.nlm.nih.gov/?term=hiv+toxoplasma+spain+prevalence>

14 results

<https://pubmed.ncbi.nlm.nih.gov/?term=toxoplasmosis+hiv+prevalence+spain>

30 results
